# Numerical Investigation on the Mechanical Properties of Vault Void Lining and Steel Plate Strengthening

**DOI:** 10.3390/ma16020789

**Published:** 2023-01-13

**Authors:** Shuai Shao, Yimin Wu, Helin Fu, Sheng Feng, Jiawei Zhang

**Affiliations:** 1 School of Civil Engineering, Central South University, Changsha 410075, China; 2 Hubei Communications Planning and Design Institute Co., Ltd., Wuhan 430051, China; 3 Tianjin Municipal Engineering Design & Research Institute, Tianjin 300392, China

**Keywords:** tunnel engineering, void lining, steel plate strengthening, numerical model, concrete-damaged plasticity, extended finite element method, failure mechanism

## Abstract

To study the mechanism of vault lining under different void heights and verify the strengthening effect of the attached steel plate, a CDP (concrete-damaged plasticity) model and the XFEM (extended finite element method) were used to construct the local numerical model of the vault void, and an experiment was carried out for verification. The strengthened structure of the steel plate was assembled with a combination of a two-component epoxy adhesive and chemical anchor bolts. Five lining models with various void thicknesses, together with their strengthened models, were evaluated. The results of the established numerical model were compared with the experimental results in terms of failure mode, vertical displacement, and load-deformation results. The results of the two numerical models were in good agreement with the experimental results, revealing the failure mechanism of the vault lining. The rigidity of the specimen after steel plate strengthening was significantly improved. When the void height was one-fourth of the secondary lining thickness, the lining cracks were reduced from 14 to 4, and the distribution width of the cracks was also reduced from 1.047 to 0.091 m after steel plate strengthening. The level of damage caused by cracking was significantly reduced, which proves the effectiveness of the surface-sticking method for steel plate strengthening.

## 1. Introduction

As of the end of 2019, the number of road tunnels in China was 19,067, a year-on-year increase of 7.5%, and the total length of road tunnels reached 18,966,600 m, a year-on-year increase of 10%. The number of extra-long highway tunnels has increased to 1175, and their total length has reached 5,217,500 m, while the number of long highway tunnels has reached 4784, and their total length is now 8,263,100 m [1].

With the rapid development of tunnel construction, the different types of lining damage have gradually increased. Among them, lining void is a common void disease. Voids change the stress state of the lining and reduce its bearing capacity [2]. As the operating time increases and the performance of the concrete lining declines, cracking or even block loss may occur, which severely affects operational safety [3]. For studying the damage characteristics of lining, many researchers have carried out loading experiments on the local lining [4,5,6]. However, they mainly focus on the shield segments, while the research on the secondary lining is less. In addition to model experiments, numerical simulation is also an effective method to study the mechanical properties of linings in tunnel engineering [7,8,9]. Meguid and Dang [10] used elastoplastic mechanics to establish a two-dimensional model and analyzed the influence of voids on the distribution of circumferential stress and the bending moment of the lining. Zhang et al. [11] tested models to study the effect of contact loss on the lining of a double-arch tunnel when the surrounding rock behind the lining formed a void and established a numerical model for verification. Numerous studies proved that voids have a great impact on tunnel lining, which is reflected in the bending moment and axial force [12,13].

However, the above studies focused on the voids caused by the absence of the surrounding rock, but the voids resulting from an insufficient lining thickness are also a common form of void. Compared with the absence of the surrounding rock, an insufficient lining thickness results in voids that not only change the lining load but also directly decrease the stiffness of the lining structure and reduce the bearing capacity of the lining [14,15]. Naotoshi Yasuda et al. [16] provided a two-dimensional elastic solution when a deep tunnel lining has a void with insufficient thickness. The results showed that the stress state of the lining changed from an axial thrust to a bending moment due to the existence of the void. Zhang et al. [17] considered the voids caused by insufficient thicknesses and established a numerical model for strengthening the lining based on a CDP model. By analyzing the influence of void location, void type, and degree on the mechanical properties of the lining, the results showed that the stiffness of the hollow lining had a weakening effect.

In this study, a loading experiment was carried out by building local specimens of vault void lining, and the mechanical characteristics and damage of the void lining with different void heights were analyzed. The experimental results were verified using models based on CDP and XFEM, and the accuracy of the two methods was analyzed through the simulation of the specimens.

## 2. Experimental Background

**Figure 1:**
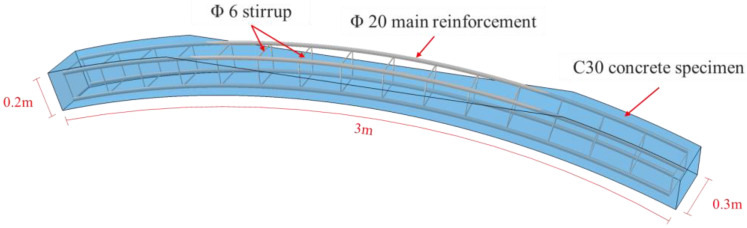
Model setup.

**Figure 2:**
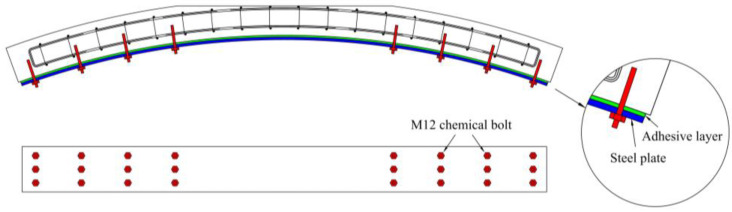
Steel plate strengthening scheme.

As shown in Figure 1, the length, width, and height of the long arc of the specimen were 3 m, 0.3 m, and 0.2 m, respectively. In the experiment, one standard condition and four void conditions were set up. As shown in Table 1, the voids were set in the vaults of the specimens and accounted for 1/4, 1/3, 1/2, and 2/3 of the lining thickness. In addition, to verify the effect of steel plate strengthening, four specimens were set up in which the lower surfaces were strengthened with steel plates under the same void conditions. The strengthening scheme is shown in Figure 2. The steel plate was 2.94 m long. Three rows, with a distance of 7.5 cm, and four equidistant columns of chemical anchor bolts were arranged on both sides of the void. The distance between the chemical anchor bolts and the edge of the steel plate was 5 cm.

**Figure 3:**
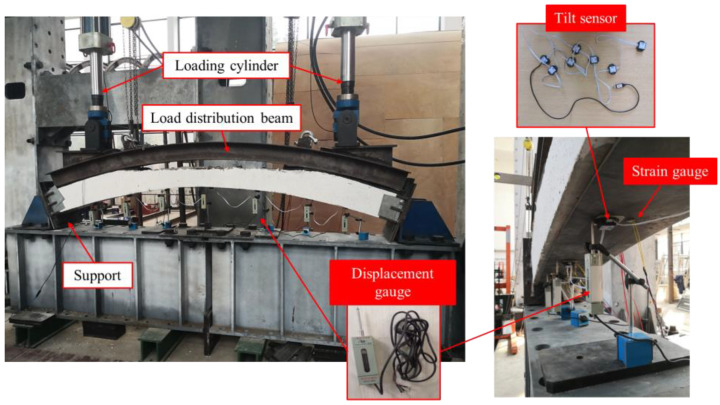
Loading device and measuring device.

In Figure 3, a diagram of the loading device and the measuring device is illustrated. In the experiment, the specimens were loaded with a 500 kN double-bar hydraulic device. In a real tunnel, the secondary lining is mainly subjected to the surrounding rock load transferred by the primary support. The vault lining is mainly subjected to the downward vertical load of the upper surrounding rock. Therefore, a rigid distribution beam was used to provide a vertical, uniform load to the specimens. The lining in the void area did not bear the load, which is the same as real-world conditions. The loading process was divided into 10 stages. The loading value of each stage was 50 kN, and the loading time of each stage was 1 min. A one minute interval was maintained between two successive loading stages. A rigid connection was used between the model and the support. Due to the lateral load and stiffness of the lining, the spandrel lining has a restraining effect on the deformation of the vault lining. This kind of restraint results in a compressive axial force and a bending moment, which is a rigid constraint. Therefore, rigid supports were used in the experiment to replace the constraint of adjacent lining on the vault lining. In order to reduce the local damage caused by the uneven contact surface at the support, a thin layer of rubber was added between the contact surface of the model and the support. 

**Figure 4:**
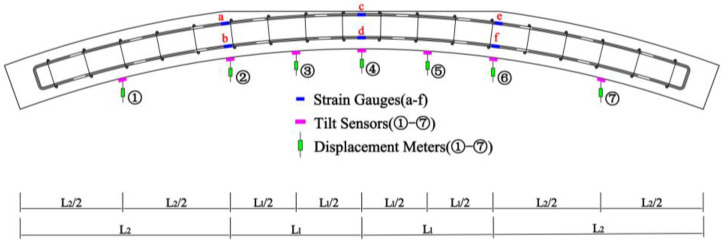
Scheme of the measuring points.

The scheme of the measuring points is shown in Figure 4. Seven measuring points were used on the lower surface of the specimens, which were distributed at the middle of the span, the edge of the void, and the middle between them. The measuring points were numbered from ① to ⑦ from left to right. Each measuring point was arranged with a tilt sensor and a displacement sensor. Strain gauges were arranged on the upper and lower layers of the rebars at the midspan and void boundary to measure the strain of the rebars, which were numbered from a to f.

## 3. Numerical Model Settings

### 3.1. Material Constitutive Model

#### 3.1.1. Constitutive Relation of Concrete

In the traditional finite element method, it is difficult to analyze the damage and fracture behavior of concrete structures because the structure is divided into adjacent small units and the unit balance equation is calculated based on the principle of minimum potential energy. By interpolating the Gaussian integral points inside the element, the value of any point in the element is solved. However, fractures and damage often cause discontinuities in the calculation. The calculation of this discontinuity is key to the effective mitigation of concrete damage and cracking.

Initially, the classical plastic theory was used to simulate the plastic deformation of concrete; however, crack occurrence and the law of crack growth cannot be defined with this theory, as the decrease in material stiffness with the degree of damage is not considered. Therefore, to more accurately simulate the damage and cracking behavior of concrete, it is necessary to consider the degradation in material properties and plastic deformation. In this study, a CDP model and the XFEM were used to describe the damage and cracking behavior of concrete, respectively.

The model was made of C30 concrete, with an elastic modulus of 3 × 10^4^ MPa, a Poisson ratio of 0.2, and a density of 2850 kg/m^3^. The elastoplasticity parameters of the CDP and the fracture parameters of XFEM were determined.

(a) CDP

The concrete-damaged plasticity (CDP) model is based on the elastoplastic damage constitutive model [18,19,20,21,22,23]. The elastoplastic damage model is a nonlinear constitutive model that combines the damage model and the elastoplastic model to describe the stress–strain relationship of concrete. In 1989, Lubliner et al. [24] proposed a new type of yield surface based on the testing of concrete material properties and proposed a single-scalar elastoplastic damage model, also known as the Barcelona model in academia. In this model, mesh size and fracture energy under compression and tension are used to modify the softening sections of the concrete due to compression and tension. However, the model of Lubliner et al. is defined in the Cauchy stress space, and therefore, shrinking the yield surface becomes problematic in the softened section of the concrete, which is not conducive to the stability of the constitutive model.

**Figure 5:**
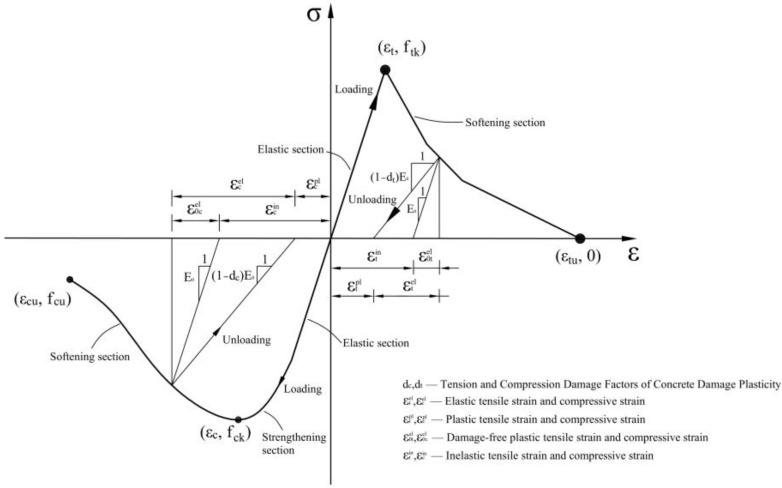
Constitutive curve of the concrete-damaged plasticity model.

Thereafter, Fenves and Lee used the theoretical framework of effective stress-space plastic mechanics, revised the yield surface proposed by Lubliner, and proposed a double scalar concrete-damaged plasticity (CDP) model [25,26,27]. The CDP model includes tensile and compressive damage variables. Using *The Code for the Design of Concrete Structures* (*GB 50010-2010, 2015 Revised Edition*), the constitutive curve of the CDP model was drawn, which is shown in Figure 5.

In this study, the CDP model was built in Abaqus. The stress–strain relationship of concrete can be described as follows:

(1) $$\sigma =\left(1-d_{t}\right)E_{c}\varepsilon$$

(2) $$d_{t}=\left\{\begin{array}{l} 1-\rho_{t}\left[1.2-0.2x^{5}\right]\;\;\;x\leq 1\\ 1-\frac{\rho_{t}}{\alpha_{t}{\left(x-1\right)}^{1.7}+x}\;\;\;\;x>1 \end{array}\right.$$

(3) $$x=\frac{\varepsilon }{\varepsilon_{t,r}},\;\rho_{t}=\frac{f_{t,r}}{E_{c}\varepsilon_{t,r}}$$

where $d_{t}$ is the damage evolution parameter of concrete under uniaxial tension; $f_{t,r}$ is the representative value of tensile strength, which can be taken as $f_{t}$_,_ $f_{tk}$_,_ or $f_{tm}$ according to, the actual structural analysis; $\varepsilon_{t,r}$ is the peak tensile strain corresponding to $f_{t,r}$; and the calculation formula is: $\varepsilon_{t,r}=f_{t,r}^{0.54}\times 65\times 10^{-6}$. $\alpha_{t}$ is the parameter value in the descending section of the concrete stress–strain curve under uniaxial tension, and the expression is $\alpha_{t}=0.312f_{t,r}^{2}$.

**Figure 6:**
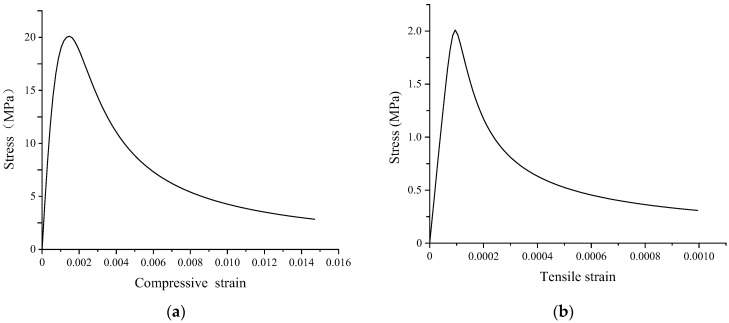
Stress–strain relationship of the concrete-damaged plastic model: (**a**) compressive stress–strain curve; and (**b**) tensile stress–strain curve.

The stress–strain relationship can be described as follows:

(4) $$\sigma =\left(1-d_{c}\right)E_{c}\varepsilon$$

(5) $$d_{c}=\left\{\begin{array}{l} 1-\frac{\rho_{c}n}{n-1+x^{n}}\;\;\;x\leq 1\\ 1-\frac{\rho_{c}}{\alpha_{c}{\left(x-1\right)}^{2}+x}\;\;x>1 \end{array}\right.$$

(6) $$x=\frac{\varepsilon }{\varepsilon_{c,r}},\;\rho_{c}=\frac{f_{c,r}}{E_{c}\varepsilon_{c,r}},\;n=\frac{E_{c}\varepsilon_{c,r}}{E_{c}\varepsilon_{c,r}-f_{c,r}}$$

where $d_{c}$ is the damage evolution parameter of concrete under uniaxial compression; $f_{t,r}$ represents the compressive strength, which can be taken as $f_{c}$, $f_{ck}$, or $f_{cm}$ according to the actual structural analysis; $\varepsilon_{c,r}$ is the peak compressive strain corresponding to $f_{c,r}$, which can be calculated by the formula $\varepsilon_{c,r}=\left(700+172\sqrt{f_{c}}\right)\times 10^{-6}$; $\alpha_{c}$ is the parameter value in the descending section of the concrete stress–strain curve under uniaxial compression and is expressed as $\alpha_{c}=0.157f_{c}^{0.785}-0.905$. The stress–strain curves and damage evolution paths are presented in Figure 6.

**Figure 7:**
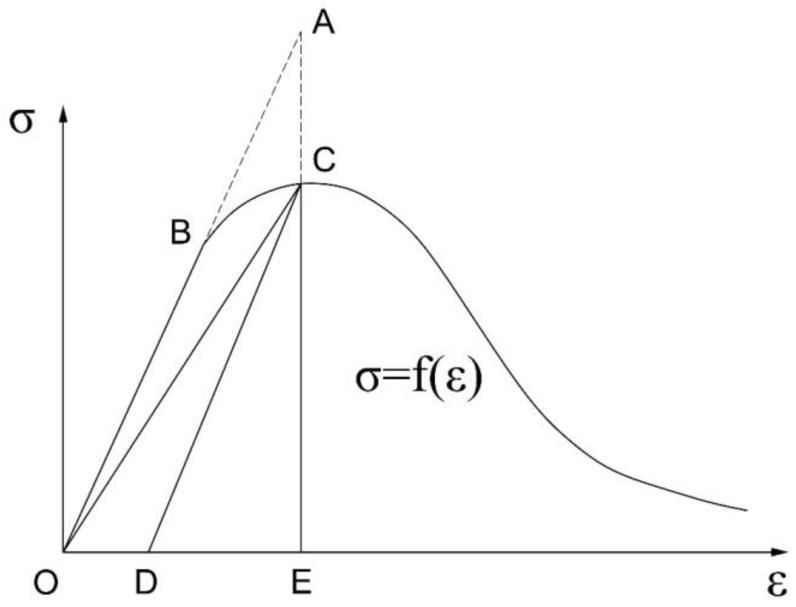
The stress–strain curve based on the concrete damage theory presented by Najar [28].

We used the damage-factor calculation method proposed by Najar in 1987 [28]. According to damage theory by Najar, the external mechanism of action in concrete essentially involves an irreversible thermodynamic process of energy dissipation. In this mechanism, the external work is transformed into elastic strain energy, plastic energy consumption, and damage expansion energy. As shown in Figure 7, according to this theory, when concrete damage is not considered, the stress–strain curve is a straight line OA.

In this case, $\sigma =E_{0}\varepsilon$, the work exerted by the external force under the nondestructive state of concrete is as follows:

(7) $$W_{OAE}=\frac{1}{2}E_{0}\varepsilon^{2}.$$

In real-world conditions, when concrete damage is present, the stress–strain curve is OBCE, and the work exerted by the external force is:

(8) $$W_{OBCE}=\int f\left(\varepsilon \right)d\varepsilon .$$

**Figure 8:**
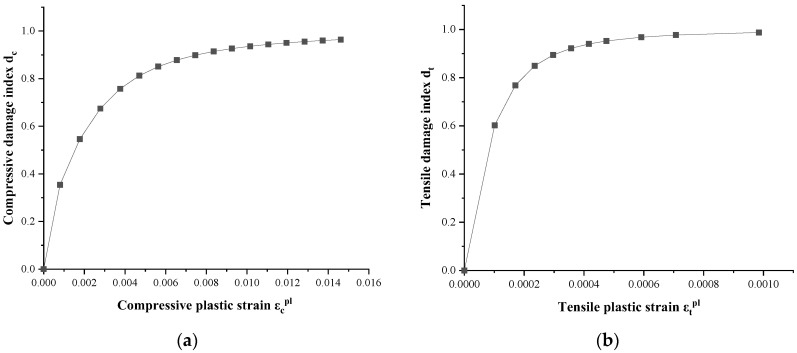
Concrete damage index: (**a**) compressive damage index d_c_ and (**b**) tensile damage index d_t_.

$W_{ABC}$ is the area difference between $W_{OAE}$ and $W_{OBCE}$. Najar used the ratio of $W_{ABC}$ to $W_{OAE}$ as the damage factor. Based on damage theory by Najar, the damage factor is defined as:

(9) $$d_{k}=\frac{W_{ABC}}{W_{OAE}}=\frac{W_{OAE}-W_{OBCE}}{W_{OAE}}=\frac{\frac{1}{2}E_{0}\varepsilon^{2}-\int f\left(\varepsilon \right)d\varepsilon }{\frac{1}{2}E_{0}\varepsilon^{2}}\;\left(k=c,t\right)$$

using the above formula, the concrete damage index was determined, and its curves for compression and tension are shown in Figure 8.

(b) XFEM

The extended finite element method (XFEM) is a method based on the partition of unity [29], level set [30], and a cohesive zone model [31,32,33]. XFEM adds a step function and an asymptotic function of the approximate displacement field of the crack tip to the approximate function of the finite element method so that it can deal with the discontinuities and singular points in the grid and is suitable for the simulation of failure and dynamic crack propagation.

**Figure 9:**
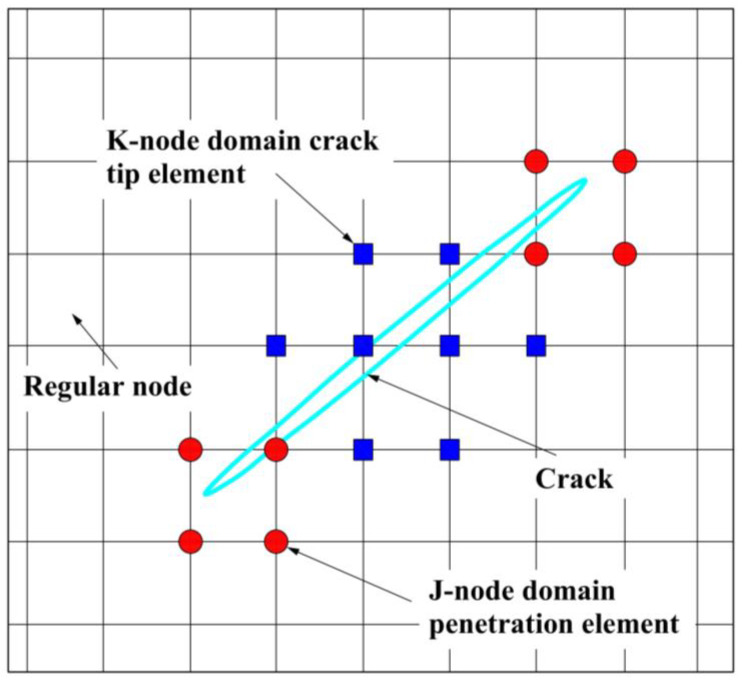
Node domain element with cracks.

Compared with the finite element method, XFEM has the following advantages: ① remeshing is not needed during the crack propagation process; ② the crack tip does not need to be densified; and ③ it does not have any data transfer problems due to having the same mesh. As shown in Figure 9, the central idea of XFEM is to introduce a step function on the crack surface based on unit decomposition to characterize the displacement function of the crack tip. XFEM discretizes the overall displacement field function as:

(10) $$\vec{U}=\sum_{I=1}^{N}N_{I}(x)\vec{u_{I}}+\sum_{j=1}^{N_{j}}N_{j}(x)H(x)\vec{a_{j}}+\sum_{k=1}^{N_{k}}N_{k}(x)\vec{b_{k}^{tip}}$$

where *N_I_*(*x*) is the commonly used nodal displacement function; *I* is the set of all the nodes of this element; $\vec{u_{I}}$ represents the finite element solution part of continuous displacement; and *J* represents the node set of the crack surface element, that is, the part that is completely penetrated by the crack but does not include the crack tip. *N_j_*(*x*) is the displacement function of the *j*-node domain. *H*(*x*) is the discontinuous jump function of the crack surface. $\vec{a_{j}}$ is the extra strengthened degrees of freedom through the element nodes. *N_k_*(*x*) is the asymptotic displacement function of the *k*-node domain and $\vec{b_{k}^{tip}}$ is the joint-strengthening degree of freedom related to the elastic asymptotic function at the crack tip.

**Figure 10:**
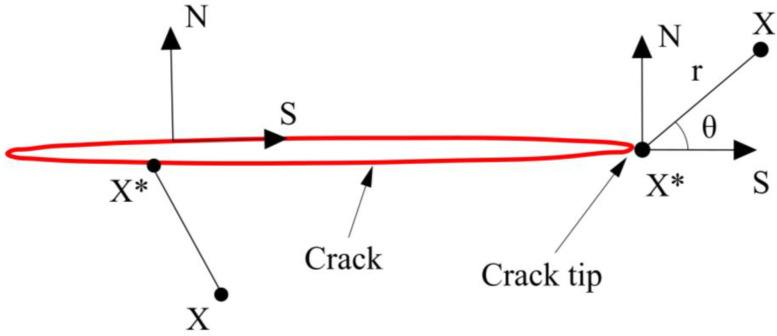
Local coordinate system of the crack tip.

The discontinuous jump function *H*(*x*) is the Heaviside function [34], reflecting the discontinuity of the crack surface displacement, as shown in Equation (11) as follows:

(11) $$H(x)=\left\{\begin{array}{l} 1\cdots \cdots \;\left(\vec{x}-{\vec{x}}^{*}\right)\cdot \vec{n}⩾0\\ -1\cdots \cdots \;\left(\vec{x}-{\vec{x}}^{*}\right)\cdot \vec{n}<0 \end{array}\right.$$

in Equation (11), ${\vec{x}}^{*}$ is the Gaussian point under investigation. ${\vec{x}}^{*}$ is the point on the crack closest to J. As shown in Figure 2, $\vec{n}$ is the outer normal unit vector, which means that the upper part of the crack surface is taken as +1 and the lower part of the crack surface is taken as −1. The asymptotic displacement function *N_k_*(*x*) characterizes the singularity of the crack tip of the isotropic material, and its expression in the local coordinate system [35] shown in Figure 10 is:

(12) $$N_{k}(x)=\left[\sqrt{r}\sin\frac{\theta }{2},\sqrt{r}\cos\frac{\theta }{2},\sqrt{r}\sin\theta \sin\frac{\theta }{2},\sqrt{r}\sin\theta \cos\frac{\theta }{2}\right]$$

(13) $$\left\{\begin{array}{l} r=\sqrt{{\left(x-x_{\mathrm{tip}\;}\right)}^{2}+{\left(y-y_{\mathrm{tip}\;}\right)}^{2}}\\ \theta =\arctan\left(\frac{y-y_{\mathrm{tip}\;}}{x-x_{\mathrm{tip}\;}}\right)-\gamma \end{array}\right.$$

where (*r*, *θ*) is the polar coordinate system in which the origin of the coordinates is the crack tip and the tangent direction of the crack tip corresponds to *θ* = 0°. *γ* is the angle between the local coordinate system of the crack tip and the global coordinate system. This formula is only applicable to straight cracks. To address the problem of hidden cracks, Belytschko [36] used the mapping method.

**Figure 11:**
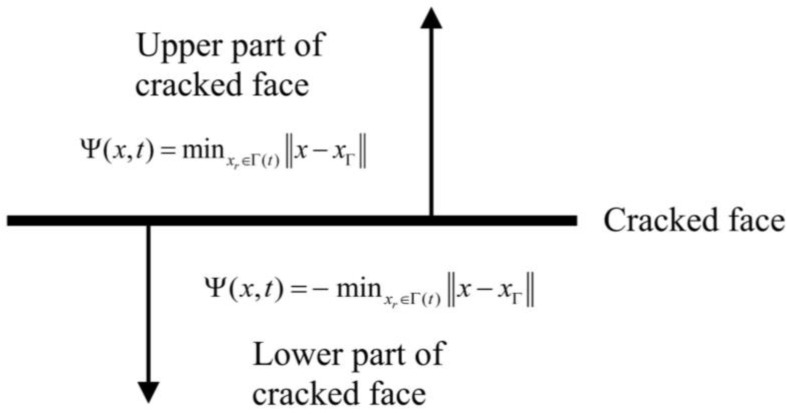
Crack level set.

To trace the crack pattern and geometrically analyze the crack propagation, the level set numerical technique was used in the expanded finite element. To study the position of the crack in the crack propagation process, the level set function is defined as $\Psi (x,t)=\pm \min_{x_{r}\in \Gamma (t)}\left\|x-x_{\Gamma }\right\|$, which is shown in Figure 11.

The crack growth can be obtained using the modified equation of Ψ(*x*,*t*) [37,38]:

(14) $$\left\{\begin{array}{l} \Psi (x,t)+F\|\nabla \Psi \|=0\\ \Psi (x,0)—\mathrm{Initial}\;\mathrm{boundary}\;\mathrm{conditions}\; \end{array}\right.$$

the extended finite element method is most widely used for the study of composite crack fracture, which makes it more suitable for the simulation of engineering fracture problems in practice. The fracture criteria for compound cracks mainly include: ① The cracks resulting from the maximum circumferential tensile stress. When the maximum circumferential stress σ_θmax_ reaches a critical value, the crack becomes unstable [39]; ② the cracks resulting from the minimum strain energy density. When the strain energy density factor Smin reaches the critical value Scr, cracks start to grow [40]; and ③ the cracks caused by the maximum energy release rate. When the critical value Gc is reached, cracks start to destabilize and expand [41,42]. 

In this study, the maximum circumferential tensile stress was used as the fracture criterion for the concrete material. By considering the circumferential tensile stress criterion, it was assumed that the direction of cracking should be such that the circumferential tensile stress reaches the maximum value, and when the stress intensity in the cracking direction reaches a critical value, the crack steadily propagates. In Abaqus, MAXPS damage was used for the concrete material, and the maximum principal stress was set to 20.1 MPa; the displacement-based cracking criterion was used for analyzing the damage evolution, and the cracking failure displacement was set to 0.0003 m.

#### 3.1.2. Constitutive Relation of Steel

**Figure 12:**
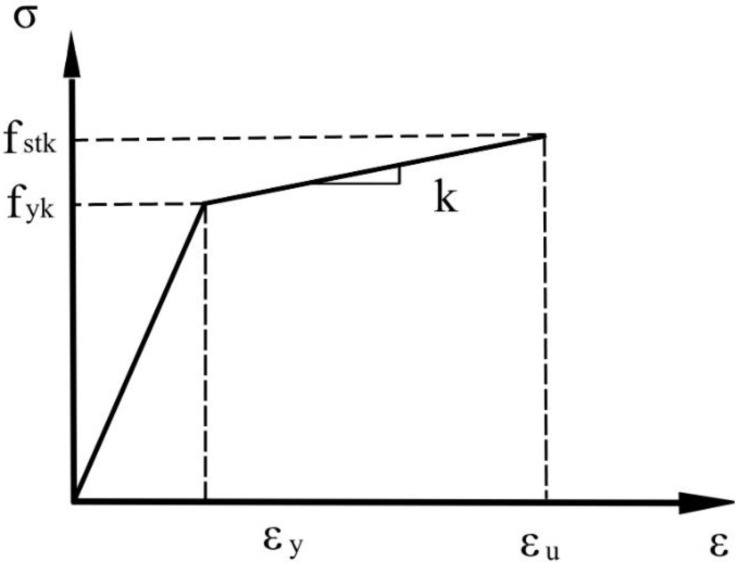
Bilinear constitutive model of steel.

As defined in the concrete design code of China (GB50010-2010), a double diagonal model was used for steel considering the hardening stage that occurs after reaching the yield strength. As shown in Figure 12, the steel was elastic before reaching the yield strength, and its slope was determined based on the elastic modulus E_0_. After reaching the yield strength, the material entered the hardening stage, and the slope of the hardening stage k was equal to 0.01 E_0_. The density of steel was set at 78.5 kg/m^3^, and Poisson’s ratio was 0.3. The grade of steel in rebars was HRB335 and the elastic modulus was 200 GPa; the standard values of yield strength f_yk_ and ultimate strength f_stk_ were 335 MPa and 455 MPa, respectively. The elastic modulus of the chemical anchor bolt was 210 GPa. The reinforcing steel plate was a Q235 steel plate. According to GB/T 700-2006, the elastic modulus of Q235 is 210 GPa, and the standard values of yield strength f_yk_ and ultimate strength f_stk_ are 235 MPa and 370 MPa, respectively.

### 3.2. Element Types and Mesh Sizes

**Figure 13:**
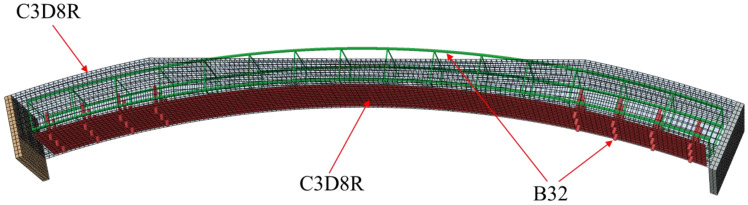
Grid diagram.

Figure 13 shows the model grid diagram. The concrete was simulated using the C3D8R element, an eight-node linear hexahedral element that controls the hourglass problem. A B32 beam element was used for rebars, and chemical anchor bolts were secondary three-node beam elements. The S4R element was adopted to simulate the steel plate, which is a four-node reduced-integration curved shell element with an hourglass control function.

### 3.3. Contact Models and Boundary Conditions

**Figure 14:**
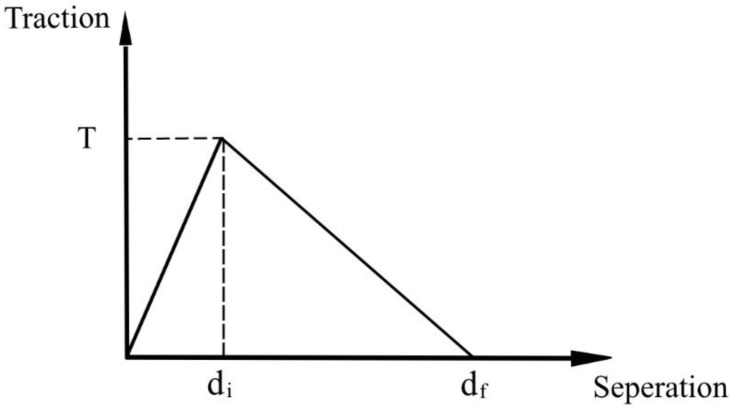
Constitutive model of structural adhesive cohesion.

To simulate the adhesive force between the steel plate and the concrete, the contact surface between these two materials was set as the cohesive surface, and the thickness of the adhesive layer was set at 10 mm. The constitutive model is shown in Figure 14. The adhesive parameters were determined based on the technical code for the safety appraisal of engineering structural strengthening materials (GB50728-2011), and they are listed in Table 2. Small slipping behavior was considered for the relative movement of the chemical anchor bolt and concrete interface. Chemical anchor bolts were attached to a steel plate, which was then embedded into the concrete through the embedded region. Surface-to-surface contact was adopted as the connection mechanism of the supports, and the friction coefficient was 0.6.

Considering the existence of the rubber pad at the boundary, the two bearings were set as grounding spring units with larger stiffness, the degree of freedom was along the *x*-axis, and the stiffness coefficient of the spring was set at 3 × 10^8^ N/m, which is close to the experimental value under the condition of ensuring the bearing stiffness.

## 4. Analysis of Simulation Results 

### 4.1. Damage

**Figure 15:**
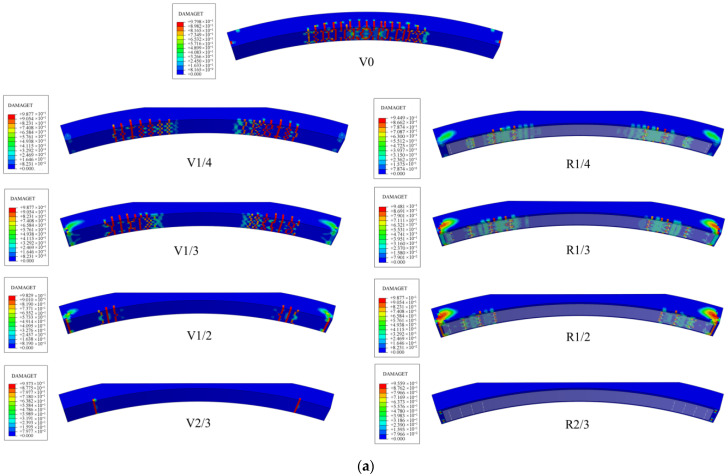

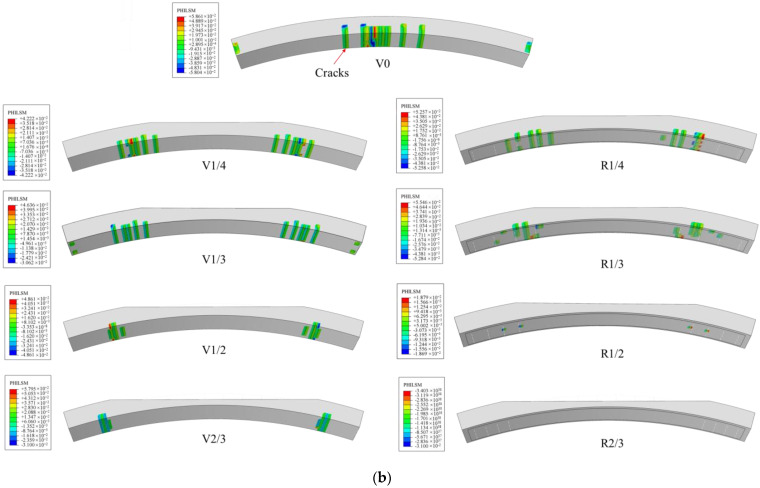
Damage pattern and crack propagation of specimens: (**a**) CDP and (**b**) XFEM.

The specimen mainly incurred tensile damage on its lower surface. Figure 15 shows the damage analysis results obtained using the CDP model and the XFEM. It can be seen that the damage results obtained by these two methods were similar. The cracks in the standard specimen were mainly distributed in the middle of the span. The stress state of the void specimen changed, and the cracking position shifted from the middle of the span to the outer edge of the void. For unstrengthened specimens, the greater the void height, the smaller the cracks on the lower surface. For the specimens with the same void height, the damage degree of the strengthened specimens was significantly lower than that of the unstrengthened specimens. The number of cracks was reduced, and the length was also reduced. This proves that the steel plate has a good strengthening effect on the lining surrounding the void. It is worth noting that the damage to the strengthened specimen was mainly concentrated around the chemical anchor bolt. This phenomenon not only proves that chemical anchor bolts have a good restraining effect on the tensile stress of the lower surface but also indicates that anchor bolts may cause a certain degree of damage to the structure during the stress process. Therefore, in the design of steel plate strengthening, an adhesive layer should be used to transfer the stress between the steel plate and the concrete surface. Chemical anchor bolts serve as additional safety stock to prevent the detachment of the steel plate.

### 4.2. Vertical Deformation

**Figure 16:**
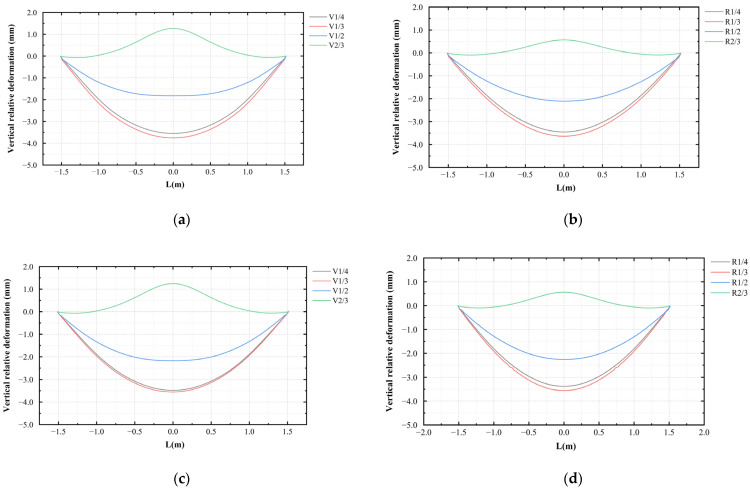
Vertical deformation: (**a**) unstrengthened specimens (CDP); (**b**) strengthened specimens (CDP); (**c**) unstrengthened specimens (XFEM); and (**d**) strengthened specimens (XFEM).

**Figure 17:**
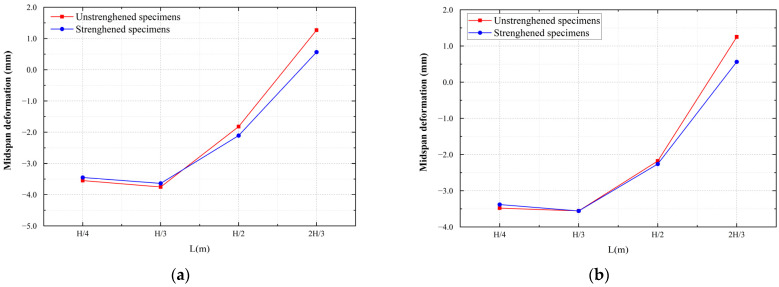
Midspan deformation: (**a**) CDP and (**b**) XFEM.

To study the deformation of the specimens during the loading process, the vertical deformation of the center line of the lower surface was determined, and the patterns are shown in Figure 16. For the unstrengthened specimens, when the void heights were H/4 and H/3, the deformation was the largest in the middle of the span and gradually decreased on both sides. When the void height was H/2, the midspan deformation was smaller and upward bulging occurred. When the void height was 2H/3 of the specimen height, the midspan bulged upwards, and a noticeable bulging pattern was observed in the specimen. Figure 17 shows the midspan deformation values for each testing condition. It can be seen from the figure that when the void height was small, the midspan deformation of the strengthened specimen was the same as that of the unstrengthened specimen. However, when the void height reached 2H/3, the steel plate effectively restricted the uplift of the specimen. The bulging in the CDP model was reduced from 1.27 to 0.56 mm, whereas the bulging in the XFEM model was reduced from 1.25 to 0.56 mm.

### 4.3. Axial Force of Rebars

**Figure 18:**
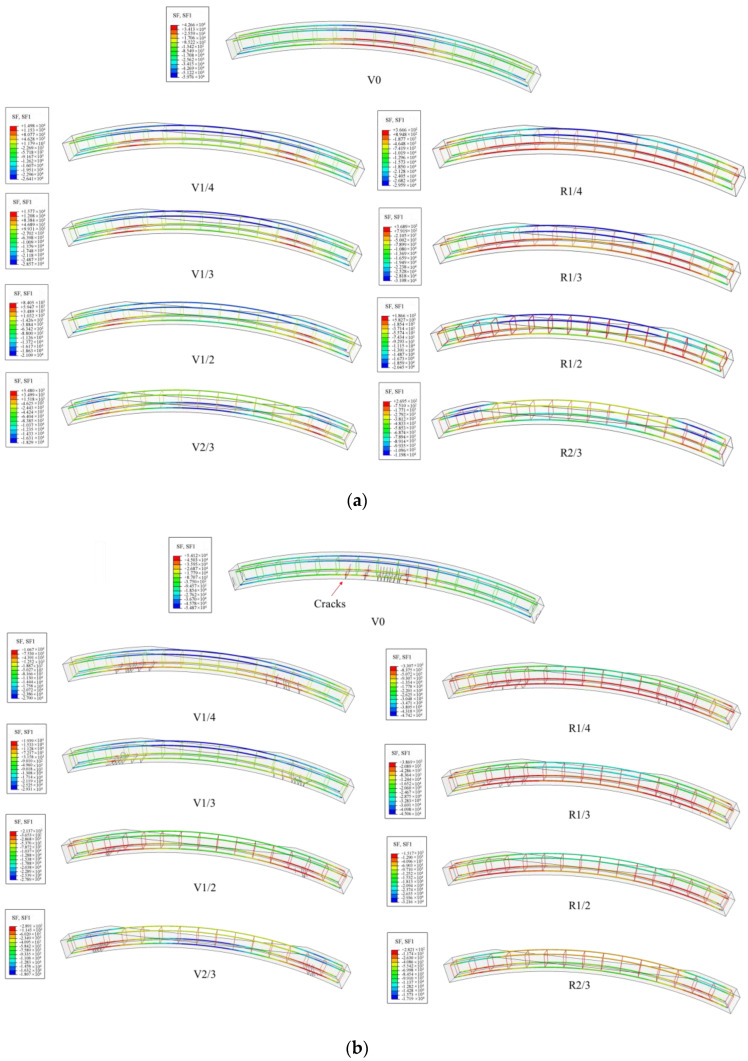
Axial forces of rebars: (**a**) CDP and (**b**) XFEM.

Due to the damage and cracking areas in the lower surface of the concrete, which caused a large deviation in the element stress value, the lower rebar was used to study the changes in the stress state of the concrete near the surface. Figure 18 shows the rebar stress of the specimens using the CDP model and the XFEM. It can be seen that the maximum tensile stress of the rebars in the standard specimen occurred in the middle of the span, while the maximum tensile stress of the rebars in the void specimens was located at the boundary of the voids.

**Figure 19:**
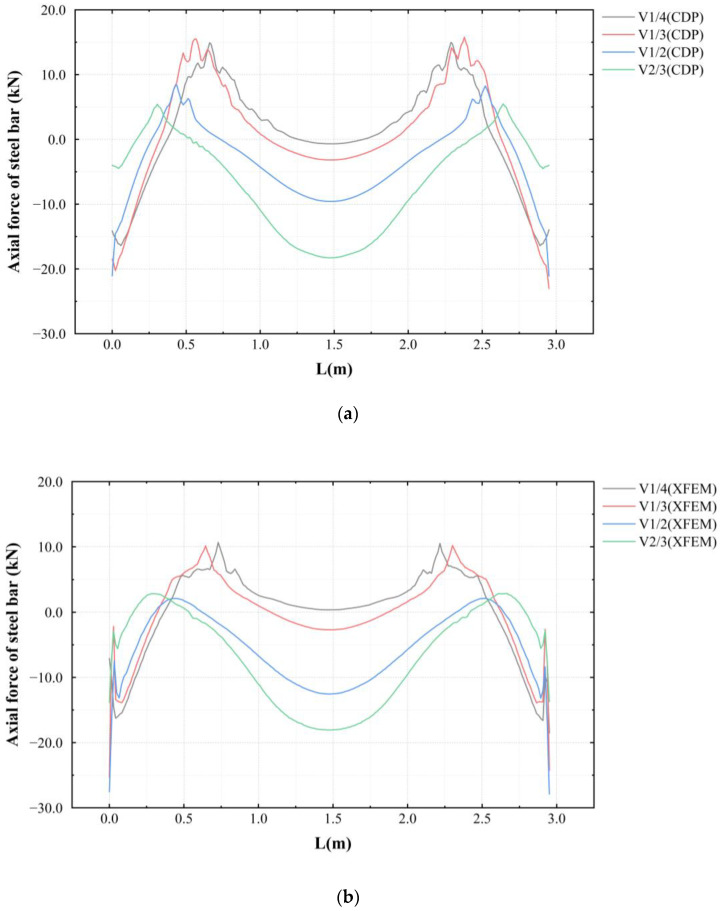
Stress of the lower rebars: (**a**) CDP and (**b**) XFEM.

Due to the damage and cracking of the concrete on the lower surface, a large fluctuation occurred in the stress of the concrete unit at the crack boundary, which is difficult to analyze. Since the lower rebars are in an elastic state, the axial force of the lower rebars was used to analyze the mechanical properties of the concrete on the lower surface. Figure 19 shows the stress on the lower rebars of the unstrengthened specimens simulated using CDP and XFEM. It can be seen from the figure that the force of the lower rebar induced an M-shaped stress pattern. The tension area was located near the void boundary, and compression occurred in the middle of the span. The tensile cracking of the concrete caused the fluctuation in the axial force of the rebar. When the void height was small, the tensile stress at the void boundary was large; by contrast, when the void height was large, the compressive stress at the vault was large. In the CDP model, when tensile damage occurred, the stiffness of the concrete degraded, which caused the lower rebars to bear more load in the area incurring concrete damage. Therefore, in the CDP model, the axial force of the lower rebars in the area with tensile damage was larger than that in the XFEM model, which is more in line with real-world conditions.

**Figure 20:**
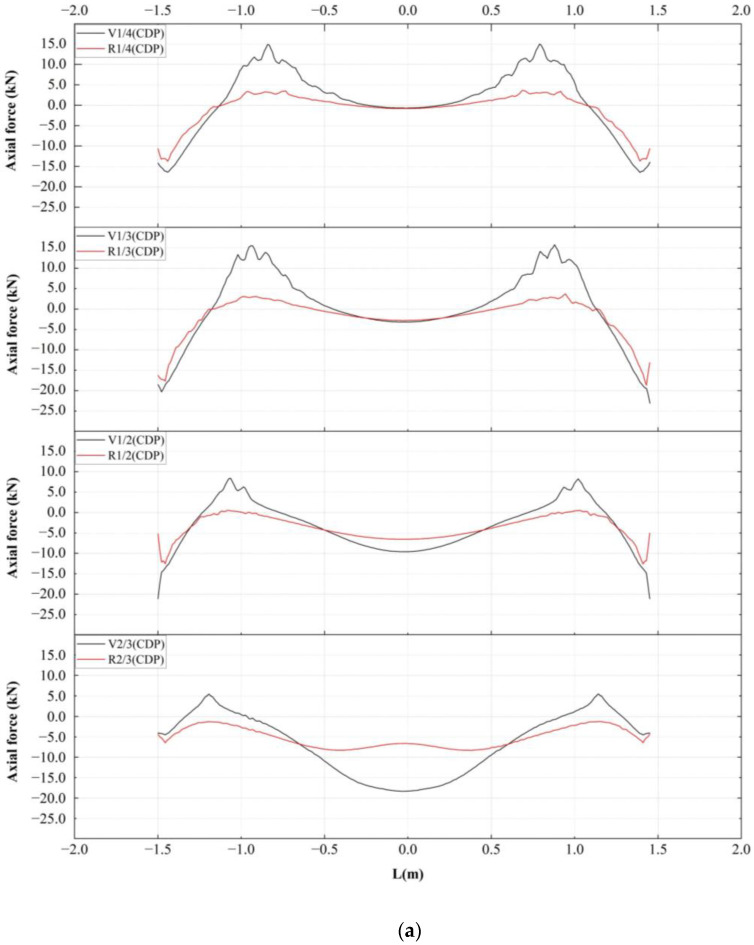

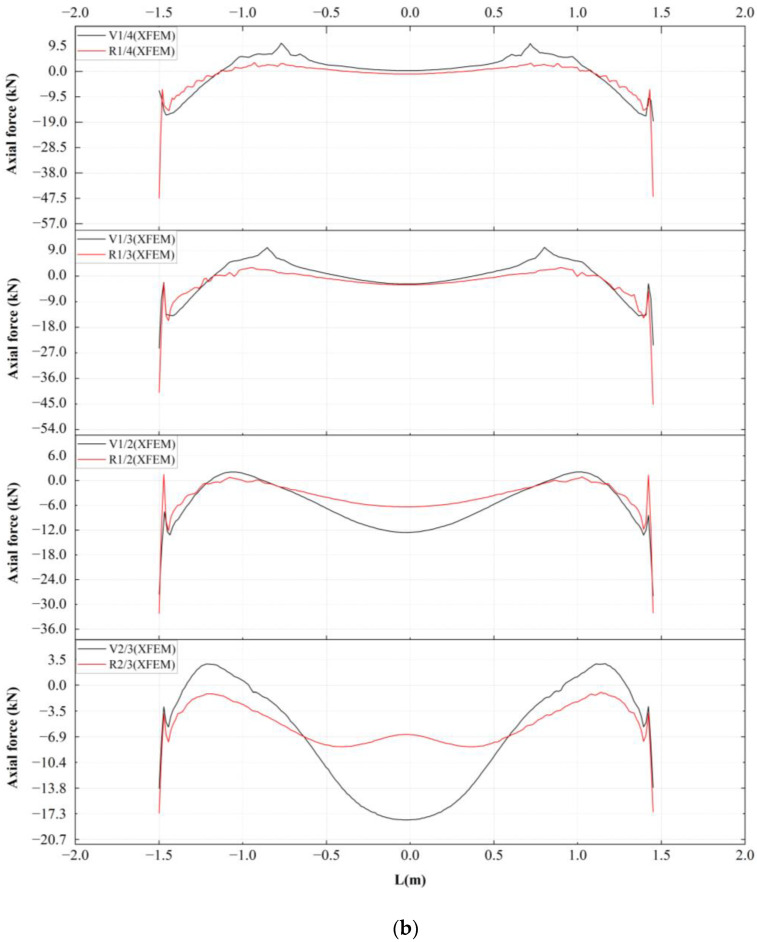
Stress comparison of lower rebars of strengthened and unstrengthened specimens: (**a**) CDP and (**b**) XFEM.

**Figure 21:**
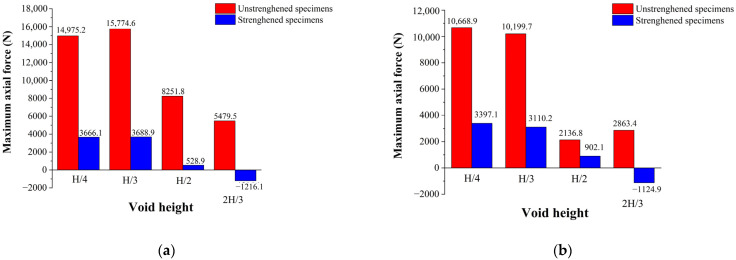
Maximum axial force of each specimen: (**a**) CDP and (**b**) XFEM.

Figure 20 shows the lower rebar stress of the strengthened specimens compared with that of the unstrengthened specimens, and Figure 21 shows the maximum axial force of each specimen. As can be seen from Figure 20, the maximum tensile axial force of the strengthened specimens was significantly lower than that of the unstrengthened specimens. In the CDP model, when the void height was H/4, the maximum tensile stress changed from 14.975 to 3.666 kN, a decrease of 75.52%. When the void height was H/3, the maximum tensile stress changed from 15.774 to 3.688 kN, showing a decrease of 76.62%. When the void heights were H/2 and 2H/3, not only was the tensile stress significantly reduced, but the compressive stress in the middle of the span was also significantly restricted, reducing by 31.77% and 54.71%, respectively. As shown in Figure 20b and Figure 21b, the calculation results of the XFEM model revealed the same trend, but since no stiffness degradation occurred, the stress concentration at the boundary was more obvious.

## 5. Comparison with Experimental Data

### 5.1. Comparison of Damage Data

**Figure 22:**
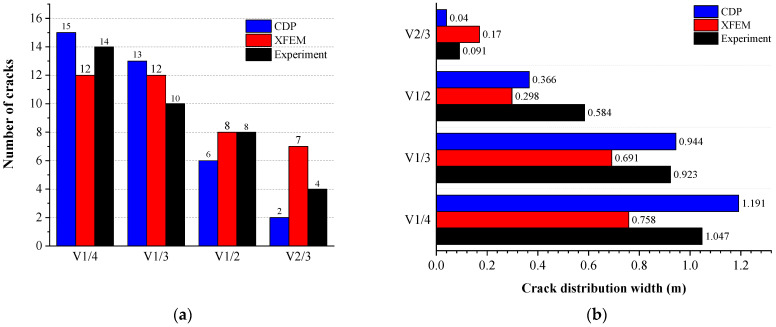
Crack behavior: (**a**) number of cracks and (**b**) distribution range of cracks.

The number and distribution range of cracks are important criteria for measuring the reliability of simulations. In order to measure the accuracy of the two methods in damage assessment, the number and distribution width of the cracks in the experiment and simulation models were extracted, which are shown in Figure 22. It can be seen from Figure 22a that the damage caused by the two simulation methods was similar to that found in the experimental results. As the void height increased, the number of lining cracks gradually decreased. The distribution range of the cracks, shown in Figure 22b, also decreased with the increase in the void height.

### 5.2. Comparison of Vertical Deformation

**Figure 23:**
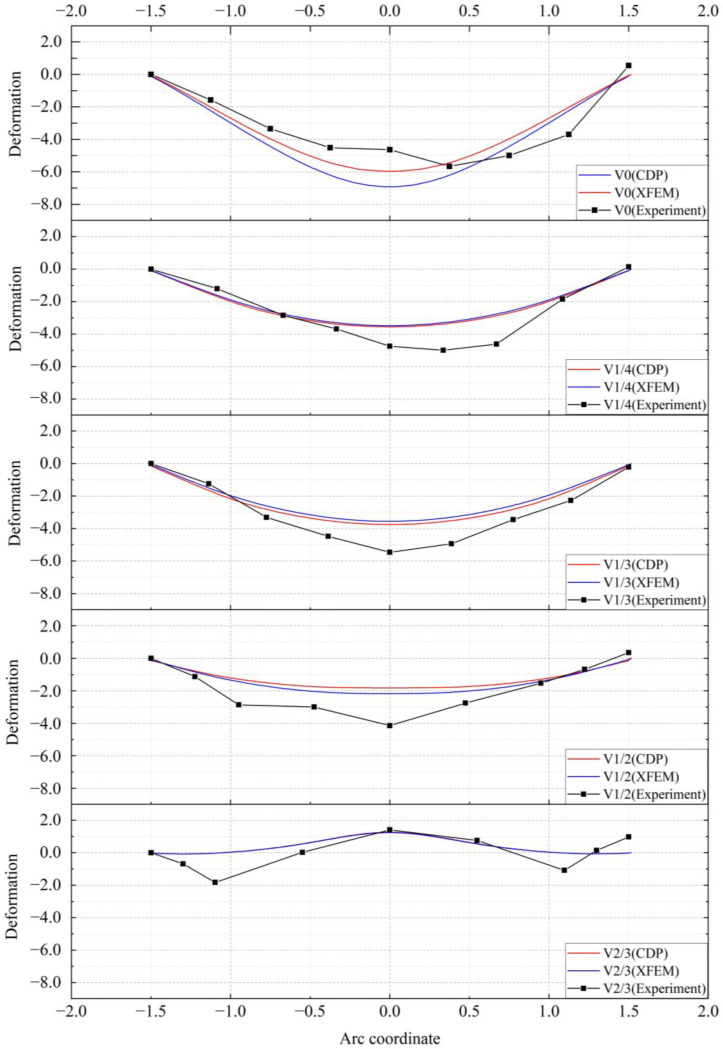
Bottom surface displacement.

As shown in Figure 23, the displacement curves of the bottom surface using the two methods were extracted with the left support as the reference point, and the results obtained in the experiment were used for comparative analysis. It can be seen from the figure that the results of the two simulation methods were almost the same except for a certain degree of error in the curves for the standard specimens. In the experiment, due to the bonding strength of the rebars, the connection strength of the stirrup and the main reinforcement, and the load-deformation behavior during loading, the deformation was slightly larger than that calculated during the simulation. However, in general, the two simulation methods were consistent with the experiment in predicting the deformation trend of the lining with different void thicknesses.

**Figure 24:**
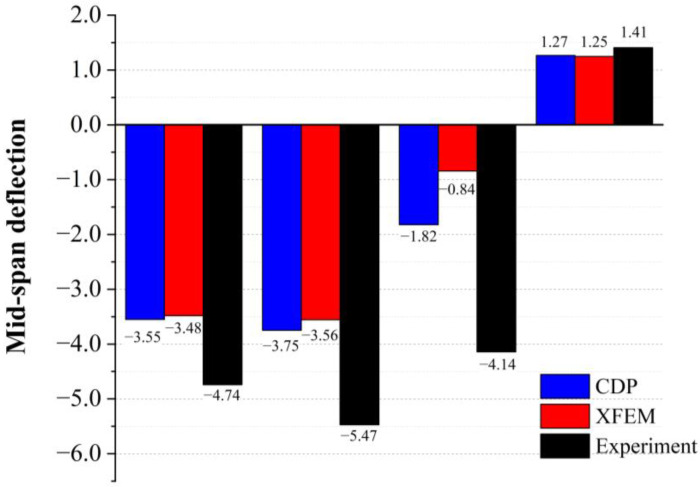
Midspan deformation.

The comparison of midspan deformation is shown in Figure 24. It can be seen from the figure that the deformation of the midspan was similar when the void thickness was H/4 and H/3. When the thickness of the void increased to H/2, the deformation was significantly reduced. When the void thickness increased to 2/3H, the midspan deformation was positive, and the midspan rose upward.

## 6. Discussion

The experimental results show that the void boundary bears the highest risk of damage. Damage at the void boundary is caused by the combined effect of a bending moment and an axial force. The bending moment of the vault lining is positive under a vertical load; that is, the lower side is under tension and the upper side is under compression. Positive bending moments lead to tensile stresses on the lower surface. When the void is large, the lining along the void shows an upward bulging behavior. The bulge decreases the positive bending moment at the void boundary and increases the axial compression force, so the tensile stress on the lower surface of the lining is decreased, and the damage on the lower surface is reduced. Therefore, based on the results obtained for the damaged area (void boundary) in the experiment, it is reasonable to believe that the principle of “the greater the void, the smaller the damage” is valid.

It can be seen from the simulation results that both the CDP model and the XFEM could simulate the characteristics of the void lining, but with some differences. In terms of damage, both methods accurately simulated the effects of the damage on the structure, the results of which were similar to the experiment results. However, in the CDP method, the cracks could not be clearly assessed by the damage value of the element, while the XFEM method directly displayed the cracks on the model. From the stress point of view, the stress of the concrete is hard to be quantitatively analyzed due to the abrupt change in the lower surface stress near the crack. Therefore, the axial force of the main reinforcement was used to represent the stress state of the specimen. In terms of the axial force of the main reinforcement, some differences exist between the two methods, mainly because in the CDP method, the degradation of material stiffness is taken into account. Once the maximum tensile stress was exceeded, the concrete stiffness entered a rapid decline phase. At this time, the concrete strength decreased, resulting in an increase in the axial force on the main reinforcement. In terms of deformation, the results obtained with the two methods were relatively similar, and the error was very small. In the experiment, due to the influence of the accuracy of double-cylinder loading and model making, the deformation value was larger than the simulation value, but the overall trend was the same.

Although both methods can simulate the damage and failure of concrete structures, they also have their shortcomings. Percentages were used in the CDP method to calibrate the lining damage, but no clear standard exists for the specific damage value obtained during cracking. The cracking behavior in the XFEM model must start at the element boundary and end at the element boundary. The model can only qualitatively analyze the crack location. Thus, the crack length, width, and other characteristics are not accurate enough, and it is difficult to calculate the cross-cracking.

## 7. Conclusions

In this study, a numerical model was established through model experiments. The CDP model and the XFEM were used to simulate the cracking of a vaulted concrete structure. In addition, model experiments under different void heights were performed to verify the lining force, deformation, and damage. The main conclusions are as follows:

1. A local model experiment was carried out to study the stress characteristics of the void vault lining. The results show that the damage to the lining caused by the void was mainly concentrated on the void boundary in the form of cracks radially distributed along the lower surface. With the increase in the void height, the damage at the void boundary gradually decreased. When the void height increased from 1/4 of the secondary lining thickness to 3/4 of the secondary lining thickness, the number of cracks decreased from 14 to 4, and the length of the damaged area decreased from 1.047 to 0.091 m. In addition, the voids caused a relative uplift of the vault position. The higher the void height was, the higher the bulge was;
2. Vault void linings with steel plate strengthening were made to verify the strengthening effect. The experimental results show that the damaged area of the lower surface of the strengthened specimen was significantly reduced, the number and length of the cracks were significantly reduced, the axial force of the lower layer reinforcement was significantly reduced, and the vault bulge caused by the void improved;
3. Numerical simulations with the CDP and XFEM models were used to verify the model experiment. Through the comparison of the results of the experiment and numerical simulations, the accuracy of the numerical simulations was verified by the damage state, vertical deformation, and axial force of rebars. The results show that the numerical results were similar to the experimental results in terms of damage and deformation. CDP and XFEM revealed high reliability in the stress analysis and damage assessment of the RC lining;
4. The advantages and disadvantages of CDP and XFEM in the simulation of structural damage in RC structures were compared and analyzed. In terms of damage expression, the damage coefficient was used in the CDP model to define the damage to the element, and the crack location was not easily identified. By comparison, an independent displacement field function was used in the XFEM model, so the cracking of concrete was more intuitive. However, using the XFEM, only the tensile pattern at the opening of the cracks was detected for damage assessment, so it is difficult to analyze the compression failure using this model. Compared with XFEM, both the damage in tension and compression were defined at the same time using the CDP method, and the concrete after the damage entered a stiffness degradation stage; thus, this model is more suitable for accurate damage assessments and stress analyses in engineering practice.

## Figures and Tables

**Table 1:** Details of the specimens.

Type	Code Name	Void Depth
Standard lining	V0	N/A
Unstrengthened lining	V1/4	H/4
	V1/3	H/3
	V1/2	H/2
	V2/3	2H/3
Strengthened lining	R1/4	H/4
	R1/3	H/3
	R1/2	H/2
	R2/3	2H/3

**Table 2:** Adhesive parameters.

Normal Stress/MPa	Shear Stress/MPa	di/mm	df/mm
50	30	0.002	0.008

## Data Availability

All data are available from the authors.
